# Rivaroxaban as an effective alternative to warfarin in a patient with atrial fibrillation, thrombophilia, and left atrial appendage thrombus: a case report

**DOI:** 10.1186/s13256-017-1249-8

**Published:** 2017-04-09

**Authors:** Michele Scarano, Matteo Casale, Cesare Mantini, Egidio Imbalzano, Cristiana Consorti, Daniela Clemente, Giuseppe Dattilo

**Affiliations:** 1 Cardiology Unit, Emergency Department, Hospital “Madonna del Soccorso”, Via Silvio Pellico n.32, 63039 San Benedetto del Tronto, Ascoli Piceno Italy; 2 Department of Clinical and Experimental Medicine, University of Messina, Messina, Italy; 3 “Gabriele D’Annunzio” University, Chieti, Italy; 4 Ospedale SS. Annunziata, Chieti, Italy

**Keywords:** Atrial fibrillation, Rivaroxaban, Thrombophilia, Thrombosis, Warfarin, Case report

## Abstract

**Background:**

Atrial fibrillation is the most common cardiac arrhythmia. It is responsible for up to 20% of all ischemic strokes. Rate control and anticoagulation are crucial for atrial fibrillation management and stroke prevention.

**Case presentation:**

We present the case of an 84-year-old Italian woman with a left atrial appendage thrombus that developed despite her use of anticoagulant therapy with warfarin for a previous pulmonary embolism. She had atrial fibrillation and heterozygosity for both factor V Leiden and methylenetetrahydrofolate reductase C677T mutation, thus creating resistance to activated protein C. Anticoagulant therapy was switched to heparin for 1 week and then to rivaroxaban. After 3 months of rivaroxaban use, the thrombus disappeared.

**Conclusions:**

This case raises the issue of the ineffectiveness of warfarin therapy in complex cases involving particular thrombophilic conditions and the possibility of using rivaroxaban as a safe and effective alternative.

## Background

Atrial fibrillation (AF) is the most common cardiac arrhythmia. Its clinical presentation spans from asymptomatic AF to cardiogenic shock or devastating cerebrovascular accident. It is responsible for up to 20% of all ischemic strokes [1]. The cornerstones of AF management are rate control and anticoagulation [2]. However, anticoagulation can be trivial in patients with some types of thrombophilias. Thrombophilias are inherited and acquired hypercoagulable conditions that increase the risk of thromboembolism and, in some cases, can lead to resistance to warfarin anticoagulant therapy [3, 4]. Direct oral anticoagulants (DOACs) are now available and can be considered alternatives to warfarin for patients with nonvalvular AF [2]. Among these, rivaroxaban, an oral factor Xa inhibitor, provides more consistent and predictable anticoagulation than warfarin [5, 6].

Here, we present the case of a woman with AF and thrombophilia who developed a left atrial appendage (LAA) thrombus while on warfarin therapy and was subsequently treated with rivaroxaban.

## Case presentation

**Fig. 1:**
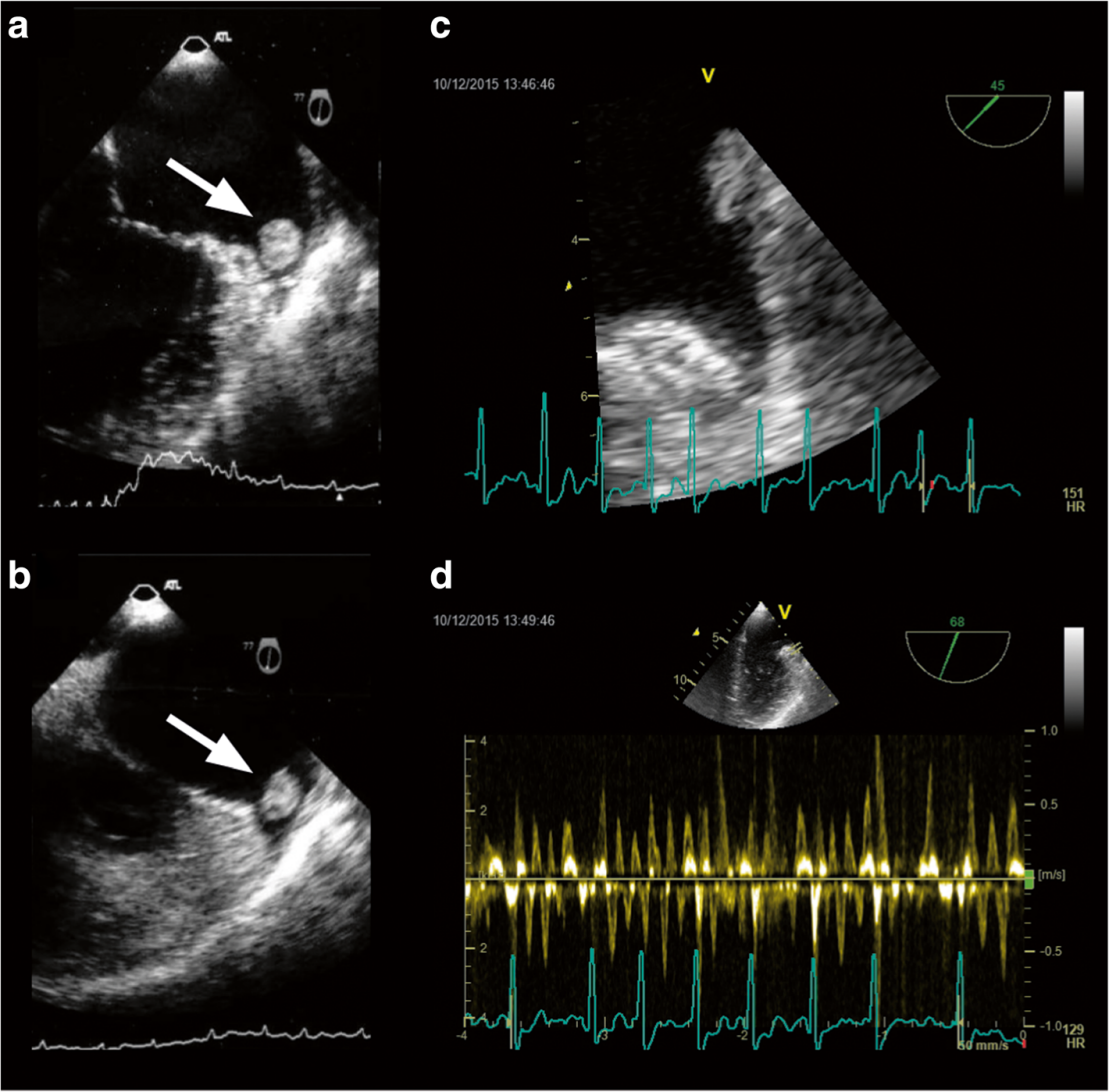
**a** Transesophageal echocardiography at diagnosis showing a mass in the left atrial appendage. **b** Transesophageal echocardiography after 7 days of treatment with intravenous unfractionated heparin showing a mass in the left atrial appendage. **c** Transesophageal echocardiography after 3 months of rivaroxaban use showing no visible mass in the left atrial appendage. **d** Doppler with high-speed blood flow in the appendage after 3 months of rivaroxaban use

An 84-year-old Italian woman presented to our hospital with a stroke and was referred to our cardiology unit for cardiologic evaluation. She had a history of hypertension, dyslipidemia, and pulmonary embolism and was using warfarin for pulmonary embolism during our evaluation: international normalized ratio (INR) 2.3; time in therapeutic range (TTR) >63%. She reported palpitations and dyspnea that started 1 week before our evaluation. An electrocardiogram showed AF with a heart rate of 96 beats per minute. Transthoracic echocardiography revealed normal left ventricle (LV) diameters and volume, left ventricular ejection fraction of approximately 60%, and a dilated left atrium (LA) with dense spontaneous echo contrast. Transesophageal echocardiography (TEE) showed a round, partially mobile, disorganized and weakly hypoechogenic mass (12 × 12 mm) that resembled a thrombus in the LAA (Fig. 1a). We examined the following in regard to thrombophilia: prothrombin time, activated partial thromboplastin time, fibrinogen, activated protein C resistance, factor V Leiden, prothrombin G20210A mutation, homocysteine, ATIII, protein C, protein S, antiphospholipid antibodies (lupus-like anticoagulant), anti-beta 2 glycoprotein I antibodies, anti-cardiolipin antibodies, factor VIII, and methylenetetrahydrofolate reductase C677T mutation. Testing revealed heterozygosity for both factor V Leiden and methylenetetrahydrofolate reductase C677T mutation, which created resistance to activated protein C [7].

We started anticoagulant treatment with unfractionated heparin administered intravenously. After 1 week of therapy, we performed a second TEE that showed that the LAA thrombus remained unchanged (Fig. 1b). Her treatment was then switched to rivaroxaban 20 mg once daily for long-term treatment and stroke prevention [8]. A third TEE performed at the 3-month follow-up visit revealed no thrombotic mass (Fig. 1c). No major or clinically relevant bleeding occurred during follow-up. At the 6-month and 12-month follow-up visits, she continued to be in good clinical condition. After thrombus resolution, because our patient was at high risk for thromboembolism and had a history of pulmonary embolism, we recommended LAA percutaneous occlusion; however, she refused this option.

For rate control, because our patient had a first diagnosed AF with mean ventricular response, we treated her with a beta blocker (bisoprolol) at low dosages (1.25 mg twice daily).

## Discussion

Thrombophilias are conditions that continue to create controversy for care providers. The case analyzed emphasizes the role of inherited thrombophilic factors in creating high risk for thromboembolism [9]. The patient described here had a thrombus in the LAA that developed despite treatment with warfarin. Two factors may have favored this: the combination of thrombophilic factors and the suboptimal TTR.

The combination of thrombophilic factors may explain, in part, the poor anticoagulant response to warfarin and the failure to reduce the LAA thrombus with heparin [1]. Heterozygosity for both factor V Leiden and methylenetetrahydrofolate reductase C677T mutation and resistance to activated protein C (a natural anticoagulant) may have created, in this particular patient with nonvalvular AF, resistance to warfarin therapy. Factor V Leiden mutation induces the resistance of factor V to cleavage by activated protein C [10]. The enzyme 5,10-methylenetetrahydrofolate reductase is involved in folate metabolism and its C677T polymorphism is associated with increased plasma homocysteine levels if the dietary folate intake is insufficient [11]. High plasma levels of homocysteine increase the risk of thrombosis by enhancing factor V activity [11]. It may be possible that, in this patient, enhanced activity of factor V combined with resistance to protein C may have favored the paradoxical procoagulant effect of warfarin as opposed to its therapeutic anticoagulant effect. Warfarin, as a vitamin K antagonist (VKA), acts indirectly on a number of coagulation factors and also on protein C, which has an important anticoagulant role in the modulation of coagulation [12].

On the other hand, warfarin may also not have provided a continuous anticoagulant effect [13]. Our patient had a TTR more than 63%, which should be improved especially considering the presence of more thrombophilic factors. The 2016 European Society of Cardiology guidelines recommend a target TTR ≥70% for patients with AF using warfarin; however, if this cannot be reached, then switching to a DOAC should be considered [2]. In this regard, and considering that the patient could not undergo percutaneous LAA occlusion, a procedure that could have lowered the risk of cardioembolic stroke [14], we decided to switch the anticoagulant therapy from warfarin to heparin for 1 week and then, for long-term therapy, to rivaroxaban, a DOAC with once-daily dosing approved for stroke prevention in patients with nonvalvular AF at high risk [15].

Cardioembolic stroke is the most serious problem in the aging population [16]. The rate of ischemic stroke in patients with nonrheumatic AF is approximately 5% per year. We cannot explain the reason for the effectiveness of rivaroxaban compared to warfarin. However, in this patient, rivaroxaban was effective not only for preventing thrombus formation but also for favoring thrombus resolution when warfarin treatment had failed [4, 17]. Optimal compliance, direct action on factor Xa, and the predictable pharmacodynamics and pharmacokinetics of rivaroxaban may have favored this higher efficacy [15].

The recently published X-TRA study, which is the first prospective, multicenter, interventional study to examine thrombus resolution with a DOAC in VKA-naïve patients or patients receiving suboptimal or ineffective VKA therapy (INR <2.0), showed that resolved or reduced thrombus after rivaroxaban treatment is consistent with LA/LAA thrombus resolution with VKA therapy reported in retrospective observational case series [18]. These results suggest that rivaroxaban may be an option for the treatment of TEE-detected LA/LAA thrombi in patients with AF or atrial flutter. It is noteworthy that in the X-TRA population, at baseline, approximately three-quarters of patients had persistent, long-standing, or permanent AF. This reflects a gap between guideline recommendations and local practice and indicates that patients diagnosed with AF are not adequately treated with an oral anticoagulant.

## Conclusions

This case raises the issue of the ineffectiveness of warfarin therapy for complex cases involving particular thrombophilic conditions and of the possibility of using rivaroxaban as a safe and effective alternative. Further investigations of the effects of the different types of anticoagulants in particular subgroups of patients with AF and inherited thrombophilias are needed to gain a better understanding of the mechanisms that can cause the ineffectiveness of VKA treatments and that can help physicians choose the best DOAC.

## Abbreviations

AF: Atrial fibrillation
DOACs: Direct oral anticoagulants
INR: International normalized ratio
LA: Left atrium
LAA: Left atrial appendage
LV: Left ventricle
TEE: Transesophageal echocardiography
TTR: Time in therapeutic range
VKA: Vitamin K antagonist
